# Translation inhibition and suppression of stress granules formation by cisplatin

**DOI:** 10.1016/j.biopha.2021.112382

**Published:** 2021-12-01

**Authors:** Paulina Pietras, Anaïs Aulas, Marta M. Fay, Marta Leśniczak-Staszak, Mateusz Sowiński, Shawn M. Lyons, Witold Szaflarski, Pavel Ivanov

**Affiliations:** a Department of Histology and Embryology, Poznan University of Medical Sciences, Poznań, Poland; b Division of Rheumatology, Inflammation, and Immunity, Brigham and Women’s Hospital, Boston, MA 02115, USA; c Department of Medicine, Harvard Medical School, Boston, MA 02115, USA

**Keywords:** Chemotherapy, Cisplatin, Stress granules, Translation initiation, Stress response

## Abstract

Platinum-based antineoplastic drugs, such as cisplatin, are commonly used to induce tumor cell death. Cisplatin is believed to induce apoptosis as a result of cisplatin-DNA adducts that inhibit DNA and RNA synthesis. Although idea that DNA damage underlines anti-proliferative effects of cisplatin is dominant in cancer research, there is a poor correlation between the degree of the cell sensitivity to cisplatin and the extent of DNA platination. Here, we examined possible effects of cisplatin on post-transcriptional gene regulation that may contribute to cisplatin-mediated cytotoxicity. We show that cisplatin suppresses formation of stress granules (SGs), pro-survival RNA granules with multiple roles in cellular metabolism. Mechanistically, cisplatin inhibits cellular translation to promote disassembly of polysomes and aggregation of ribosomal subunits. As SGs are in equilibrium with polysomes, cisplatin-induced shift towards ribosomal aggregation suppresses SG formation. Our data uncover previously unknown effects of cisplatin on RNA metabolism.

## 1. Introduction

Cisplatin [cis-diammine-dichloroplatinum(II)] (CisPt) is a leading antineoplastic platinum-based compound that is widely used to treat roughly 20 distinct tumor types [1]. The clinical benefits of CisPt as an antiproliferative and cytotoxic agent have been recognized for nearly 45 years [2]. While highly effective as a chemotherapeutic agent, CisPt causes a range of side effects including nephrotoxicity, ototoxicity, myelosuppression, gastrotoxicity and allergic reactions [3]. It is assumed, that its closely related analogs carboplatin and oxaliplatin share with CisPt a proposed mechanism of action as DNA-damaging agents, although all compounds demonstrate a difference in the spectrum of toxicities [4]. The cytotoxicity of CisPt is primarily explained by its ability to interact with N7-sites of purine bases in DNA, which promotes formation of both DNA-DNA inter- and intra-strand crosslinks [5, 6]. In turn, such crosslinks distort DNA duplex structures and create CisPt-induced nuclear lesions, the extent of which grossly correlates with extent of cytotoxicity [7]. The CisPt-induced nuclear lesions are proposed to be recognized by different DNA damage proteins or their complexes, which bind to physical distortions on the DNA. They signal then to downstream effectors that promote cascade of signaling events culminating in apoptosis [8].

For decades, it is generally postulated that DNA is a preferential and primary molecular target of CisPt and different types of DNA lesions (monoadducts, inter- and intra-strand crosslinks) trigger DNA damage responses in cells treated with platinum drugs [9]. In agreement with this model, cells deficient in DNA repair are more sensitive to CisPt [10]. However, in enucleated cells CisPt-induced apoptosis occurs independently of DNA damage [11]. Also, less than 1% of the intracellular CisPt is covalently bound to DNA and there is poor correlation between the sensitivity of cells to the drug and the extent of DNA platination [12]. Moreover, other studies have challenged the DNA-platination model suggesting that CisPt cytotoxicity originates from disrupting RNA processes including induction of ribosomal biogenesis stress [13], inactivation of splicing [14], inhibition of cellular translation [15,16] and targeting telomeric RNA [17].

Stress granules (SGs) are non-membranous cytoplasmic entities consisting of mRNAs and proteins that form upon cellular exposure to various biotic and abiotic stresses. SGs play critical roles in the Integral Stress Response coordinating multiple cellular processes aimed at promoting cell survival [18]. In cancer cells, SGs confer cytoprotection against chemotherapy, radiotherapy and hostile tumor microenvironment [19]. SGs promote cell survival on multiple levels. SGs block apoptotic pathways by acting as signaling hubs to rewire signaling cascades and act as platforms to re-program cellular translation to conserve energy and redirect that energy to repair stress-induced damage [20]. Additionally, tumor cells promote SG formation to enhance cancer cell fitness and resistance to chemotherapy induced stress thus making SGs potential targets for anti-cancer therapy [19].

Under stress, two major regulatory pathways contribute to SG assembly and modulate protein synthesis by targeting translation initiation [21]. The first pathway targets eukaryotic initiation factor 2 alpha (eIF2α), a component of the eIF2/GTP/tRNAiMet ternary complex that delivers initiator tRNAiMet to the 40S ribosomal subunit. eIF2α is phosphorylated at serine 51 (S51) by one of several stress-activated eIF2α kinases (PKR, PERK, GCN2 and HRI) which inhibits efficient GDP-GTP exchange, prevents the assembly of the ternary complex, and thus inhibits translation initiation (discussed in details in Ref. [22]). The second pathway regulates the assembly of the cap-binding eIF4F complex, consisting of eIF4E, eIF4G and eIF4A, controlled by the PI3K-mTOR (mammalian target of rapamycin) kinase cascade. Under optimal conditions, mTOR constitutively phosphorylates its downstream target, eIF4E-binding protein 1 (i.e., eIF4E-BP1 (4E-BP1)) preventing its interaction with eIF4E. Stress-induced inactivation of mTOR leads to the dephosphorylation of 4E-BP1. Dephosphorylated 4E-BP1 prevents the assembly of eIF4F leading to inhibition of translation initiation (reviewed in Ref. [23]).

Here, we demonstrate that CisPt affects multiple aspects of mRNA translation by several non-overlapping mechanisms. First, it inhibits translation initiation by promoting 4E-BP1 dephosphorylation and eIF2α phosphorylation. Second, it targets ribosomes and inhibits SG formation in a concentration- and time-dependent manner. CisPt prevents ribosome engagement into translation complexes by inhibiting translation initiation and promoting small ribosomal 40S subunit aggregation in cytosol (CisPt foci). The composition and mechanisms of assembly of CisPt foci are different from canonical SGs [24,25]. They fail to recruit polyadenylated (poly(A)) mRNAs and lack some SG-associated translation initiation factors. In contrast to SGs, CisPt foci are long lasting, less dynamic and largely unaffected by pharmacological manipulations of polysomes, translating fraction of ribosomes that form equilibrium with canonical SGs. Formation of CisPt foci sequesters 40S ribosomal subunits and, thus, decreases the number of translating ribosomes, which consequently affecting the formation of SGs. These data demonstrates that cisplatin has pleiotropic effects on cellular RNA metabolism that my contribute to pro-apoptotic effects of CisPt on cancer cells.

## 2. Materials and methods

### 2.1. Cell culture

Human osteosarcoma cells (U2OS, ATCC® HTB-96™), human cervix cancer cells (SiHa, ATCC® HTB-35™), human uterus cancer cells (MES-SA, ATCC® CRL-1976™), human cervix cancer cells (HeLa, ATCC® CCL-2™) mouse embryonic fibroblasts (MEFs) with/without S51A mutation of eIF2*α*, and Dcp1-YFP expressing U2OS cells were grown in Dulbecco’s Modified Eagle Medium with 4.5 g/l D-glucose (DMEM, Gibco) supplemented with 10% fetal bovine serum (Sigma-Aldrich) and Penicillin-Streptomycin cocktail (Sigma-Aldrich). HAP1 cells: (a) parental (PAR), (b) eIF2α (S51A), (c) ΔHRI, (d) ΔGCN2, (e) ΔPKR, (f) ΔPERK (Horizon Discovery, UK) grown in Iscove’s Modified Dulbecco’s Medium (IMDM, Gibco) supplemented as described for DMEM. Kinase-negative HAP1 cells were verified by sequencing (Fig. S6).

### 2.2. Antibodies

Anti-G3BP1 (sc-81940; 1:200 dilution for IF), anti-eIF4G (sc-11373; 1:200 dilution for IF, 1:1000 for WB), anti-eIF3b (sc-16377; 1:200 dilution for IF), anti-FXR1 (sc-10554, 1:200 dilution for IF), anti-TIAR (sc-1749; 1:1000 dilution for IF), anti-TIA-1 (sc-1751; 1:1000 dilution for IF), anti-HuR (sc-5261; 1:200 dilution for IF), anti-PABP (sc-32318; 1:100 dilution for IF), anti-p70 S6 kinase (sc-8418, 1:200 dilution for IF) and anti-TRAF2 (sc-2345, 1:200 dilution for IF) were purchased from Santa Cruz Biotechnology (US). Anti-total-eIF2α (#2103, 1:1000 dilution for WB), anti-non-Phospho-4E-BP1 (#4923, 1:1000 dilution for WB), anti-UPF1 (#9435; 1:200 dilution for IF), anti-P-rpS6 (#2211; 1:1000 dilution for WB) and anti-Rsk2 (#5528; 1:200 dilution for IF) were purchased from Cell Signaling Technology. Anti-Tubulin *α* (66031-1-Ig; 1:1000 dilution for WB), anti-Caprin 1 (15112-1-AP; 1:200 dilution for IF), anti-ABCE1 (14032-1-AP, 1:200 dilution for IF) and PELO (10582-1-AP, 1:200 for IF) were purchased from Protein Technology Group. Anti-ph-eIF2α (Ab32157; 1:1000 dilution for WB) was purchased from Abcam. Anti-Puromycin (MABE343; 1:200 dilution for IF; 1:1000 dilution for WB) was purchased from Millipore. The secondary antibodies for WB, i.e., Peroxidase AffiniPure Donkey Anti-Mouse IgG (cat. 715-035-150) and Peroxidase AffiniPure Donkey Anti-Rabbit IgG (711-035-152) were purchased from Jackson ImmunoResearch. The secondary antibodies for IF included Cy™2 AffiniPure Donkey Anti-Mouse IgG (cat. 715-225-150), Cy™3 AffiniPure Donkey Anti-Rabbit IgG (711-165-152) and Alexa Fluor® 647 AffiniPure Bovine Anti-Goat IgG (805-605-180) and were purchased from Jackson ImmunoResearch.

### 2.3. Anticancer drugs and chemical compounds

Cisplatin was purchased from BioTang Inc. Cisplatin was prepared directly in DMEM and kept at 4 °C. Vinorelbine was purchased from BioTang Inc. Oxaliplatin (commercially available anticancer drug, solution 5 mg/ml) was purchased from Teva Pharmaceuticals, Poland. Carboplatin (commercially available anticancer drug, solution 10 mg/ml) was purchased from Actavis Group PTC, Iceland. Sodium arsenite, puromycin, cycloheximide, and emetine were purchased from Sigma-Aldrich.

### 2.4. Immunofluorescence microscopy

The immunofluorescence technique was done as previously described [26]. Shortly, cells were fixed in 4% paraformaldehyde (Sigma-Aldrich) and permeabilized in cold methanol (− 20 °C). Then, cells were incubated with blocking buffer (5% Horse Serum in PBS) for 1 h. Cells were incubated with primary antibodies overnight and with secondary antibodies for at least 1 h and washed twice with PBS in between incubations. Hoechst 33258 (Sigma-Aldrich) or DAPI (Sigma-Aldrich) was used together with the secondary antibodies in order to stain the nuclei. Cover slips with cells were mounted in polyvinyl mounting medium. Cells were imaged using an Eclipse E800 Nikon or AxioImager Carl Zeiss microscopes and photographed with either a SPOT CCD or a Pursuit CCD camera (both from Diagnostic Instruments) using the manufacturer’s software. The images were analyzed and merged using Adobe Photoshop CC.

### 2.5. Fluorescence in vitro hybridization (FISH)

10^5^ cells grown on coverslips were fixed in 4% formaldehyde in PBS (10 min) and subsequently permeabilized in 96% cold methanol (10 min). PerfectHyb™ Plus Hybridization Buffer (Sigma-Aldrich, H7033) was used to block samples (15 min at 52 °C) and hybridize the probe (synthetic oligo-dT_40_ labeled with cy3 or cy5, 18S rRNA: 5’-TTGAGACAAGCATATGCTACTGGC-cy3 and 5.8S rRNA: 5’-TCCTGCAATTCACATTAATTCTCGAGCTAGC-cy3) for 1 h at 52 °C. Then, samples were washed three times with 2 × SSC (the first time with pre-wormed and subsequent times with room temperature buffer) and one time with PBS. The primary and secondary antibodies with DAPI were applied (45 min each). Finally, coverslips with cells were washed twice with PBS and mounted in polyvinyl mounting medium.

### 2.6. Western blotting

Cells were grown in 6-well plates until 80% confluence. They were washed with HBSS buffer and solubilized in the lysis buffer (5 mM MES, pH 6.2%, and 2% SDS), followed by 2 × 2 min sonication at 4 °C. Lysates were denatured in a boiling water and cooled to room temperature. Proteins were precipitated in 60% acetone at − 20 °C overnight. Lysates were then centrifuged (13,500 rpm, 4 °C, 15 min) and supernatant was carefully removed and discarded. Pellets were dissolved in 1 × Laemmli loading buffer, proteins were separated in 4–20% SDS-PAGE gels (BioRad) and transferred to nitrocellulose membranes using Trans-Blot® Turbo™ system (BioRad). After 1 h blocking in 2% milk in TBS-Tween, membranes were incubated with primary and secondary antibodies for a minimum 1 h (membranes were also washed 5x after each type of antibodies). Finally, HRP-conjugated secondary antibodies were detected with SuperSignal West Pico Chemiluminescent Substrate (Thermo-Scientific) according to the manufacturer instruction.

### 2.7. Quantification of SGs

The percentage of stress granules in a cell population was quantified by manual counting of approximately 700 cells with/without stress granules using Adobe Photoshop CC. Quantification of band intensity in WB technique was done using ImageJ software.

### 2.8. Polysomes profiles

Cells were washed with cold HBSS, scrape-harvested directly into lysis buffer (10 mM HEPES pH 7.5, 125 mM KCl, 5 mM MgCl_2_, 1 mM DTT, 100 μg/ml cycloheximide, 100 μg/ml heparin, 1% NP40 made in DEPC-treated water), supplemented with RNasin Plus inhibitor (Promega) and HALT phosphatase and protease inhibitors (Thermo Scientific). Lysates were rotated at 4 °C for 15 min, cleared by centrifugation for 10 min at 12,000 *g*, and supernatants loaded on pre-formed 17.5–50% sucrose gradients made in gradient buffer (10 mM HEPES pH 7.5, 125 mM KCl, 5 mM MgCl_2_, 1 mM DTT). Samples were centrifuged in a Beckman SW140 Ti rotor for 2.5 h at 35,000 rpm, then eluted using a Brandel bottom-piercing apparatus connected to an ISCO UV monitor, which measured the eluate at OD 254.

### 2.9. Fluorescence recovery after photobleaching (FRAP)

U2OS stably expressing GFP-G3BP1 were plated the day prior the experiment. Cells were stressed as indicated and 30 min before starting the experiment cells were transferred to the FRAP chamber (37 °C, 5% CO_2_, humidified). 3 frames were collected before bleaching and 20 after, all with an interval of 5 s in-between. The photobleaching beam was positioned directly over each SG, and laser power were turn to 100% of the power to perform bleaching.

### 2.10. Ribopuromycylation assay

Ribopuromycylation assay was modified from Ref. [27], as described in Ref. [28]. In brief, 5 min before fixation, puromycin (Sigma-Aldrich) was added to a final concentration of 5 μg/ml, respectively, and the incubation continued for 5 min. Cells were then lysed subjected to either western blotting or immunofluorescence using anti-puromycin antibody (both techniques as described above). Cells without puromycin treatment were used as negative controls.

### 2.11. m^7^GTP-sepharose pulldown assay

U2OS cells grown on 10-cm dishes were lysed in 0.5 ml of lysis buffer (Tris–HCl pH7.4, 100 mM NaCl, 1 mM ethylenediaminetetraacetic acid, 0.5% NP-40, supplemented with a protease inhibitors), and centrifuged for 15 min at 13 000 rpm at 4 °C. The supernatant containing 1 mg of total protein was transferred to a clean tube and incubated with prewashed 15 μl suspension of m^7^GTP-sepharose (GE Healthcare) for 2 h at 4 °C with rotation. The beads were washed extensively with the lysis buffer and cap-bound materials were eluted by boiling in 60 μl of 2 × Laemmli’s sample buffer supplemented with 100 mM DTT.

### 2.12. Statistical analysis

Statistical analysis was done using GraphPad software (Prism). For all calculation uncoupled *t*-test was used and the statistical significance was demonstrated by numbers of stars (ns P > 0.05, * P ≤ 0.05, ** P ≤ 0.01, *** P ≤ 0.001, **** P ≤ 0.0001).

## 3. Results

### 3.1. Cisplatin induces formation of SG-like cytoplasmic foci

**Fig. 1.**
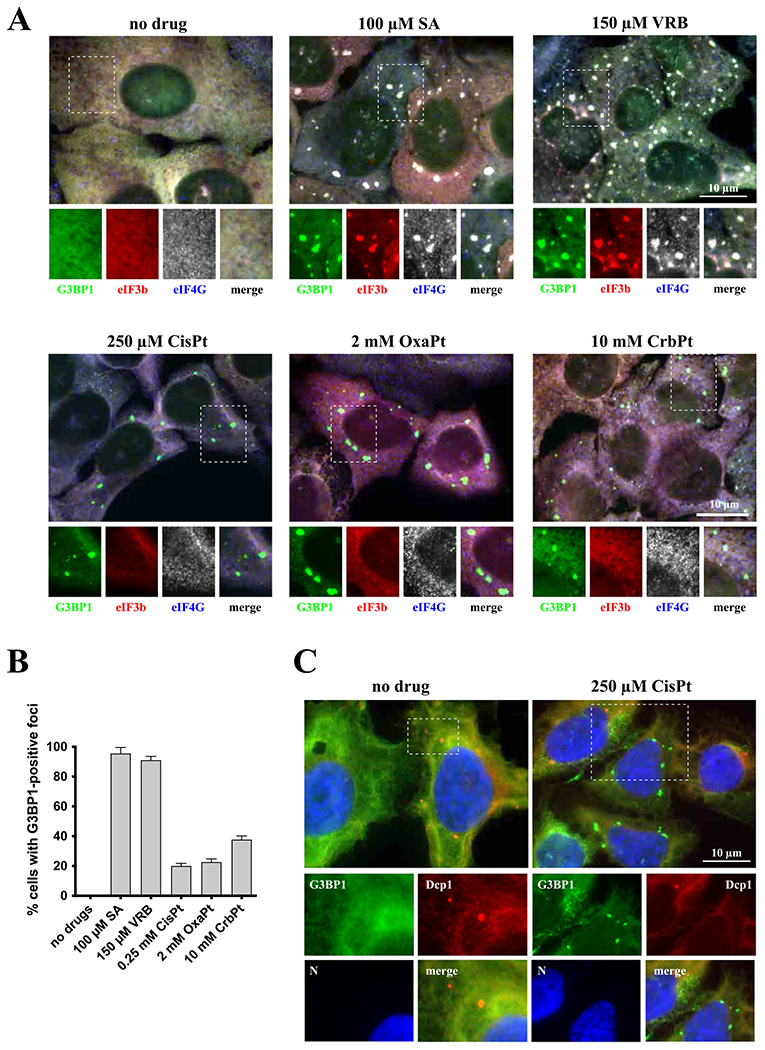
Platin-based drugs induce cytoplasmatic granules formation. (*A*) Formation of cytoplasmic granules. U2OS cells were stressed with sodium arsenite (SA, 100 μM) and vinorelbine (VRB, 150 μM) for 1 h, and with cisplatin (CisPt, 250 μM), oxaliplatin (OxaPt, 2 mM), and carboplatin (CrbPt, 10 mM) for 4 h. Unstressed U2OS cells were used as control (no drug). After treatment, cells were fixed and stained for stress granules markers: G3BP1 (green), eIF3b (red) and eIF4G (blue, shown as grey). Boxed region is shown enlarged with colors separated below each image. The size bar represents 10 μm. (*B*) Quantification of cytoplasmatic G3BP1-positive foci in U2OS cells (as shown in Fig. 1A). Data were analyzed using unpaired Student’s *t*-test, N = 3. (*C*) Detection of P-body marker Dcp1 in U2OS cells stressed with cisplatin (CisPt, 250 μM). Control, unstressed, population of U2OS cells was used as control (no drug). After treatment, cells were fixed and stained for G3BP1 (green), Dcp1 (red) and Hoechst (blue). Boxed region is shown enlarged with colors separated below each image; all colors (RGB) are merged in the main image. The size bar represents 10 μM.

**Fig. 2.**
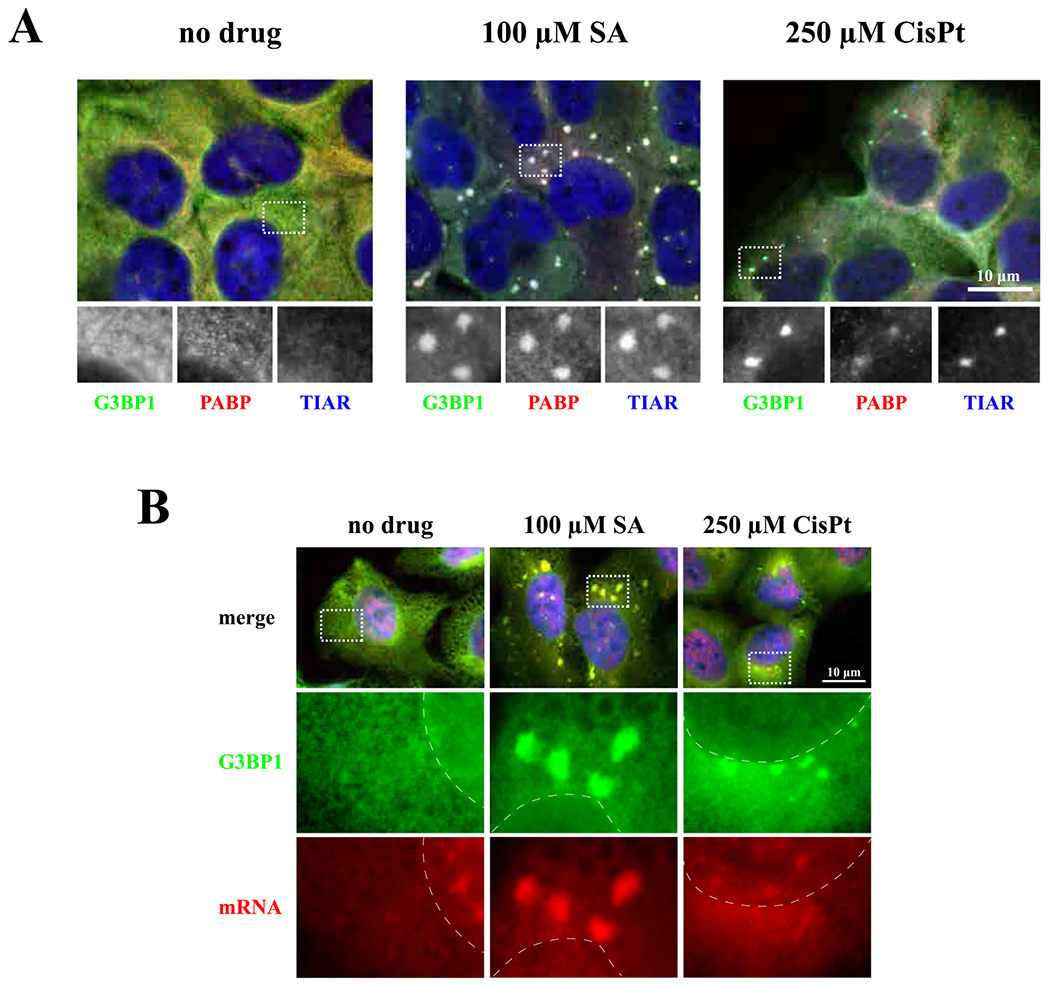
Detection of typical stress granules marker, poly(A)-binding protein (PABP), and mRNAs. (*A*) CisPt-induced foci contain PABP. One population of U2OS cells were used as unstressed control (no drug). Cells were stressed with sodium acetate (SA, 100 μM) or cisplatin (CisPt, 250 μM), for 1 h and 4 h, respectively. Then, cells were fixed and stained for G3BP1 (green), PABP (red) and TIAR (blue). All channels were demonstrated in grey in box region. The size bar represents 10 μm. (*B*) CisPt-induced foci do not contain mRNA. U2OS cells were treated with sodium arsenite (SA, 100 μM) for 1 h and cisplatin (CisPt, 250 μM) for 4 h (control cells, untreated, no drug). Cells were fixed and stained for G3BP1 and mRNA using FISH technique (G3BP1 – green – cyanine 2, mRNA – red – cyanine 3 fused with the anti-biotin secondary antibodies; in situ hybridization was done using oligo-dT40 probe against polyadenylated mRNA). Nuclei were visualized with Hoechst staining (blue). Boxed region is shown enlarged below each image, dotted line represents boundaries of nuclei.

**Fig. 3.**
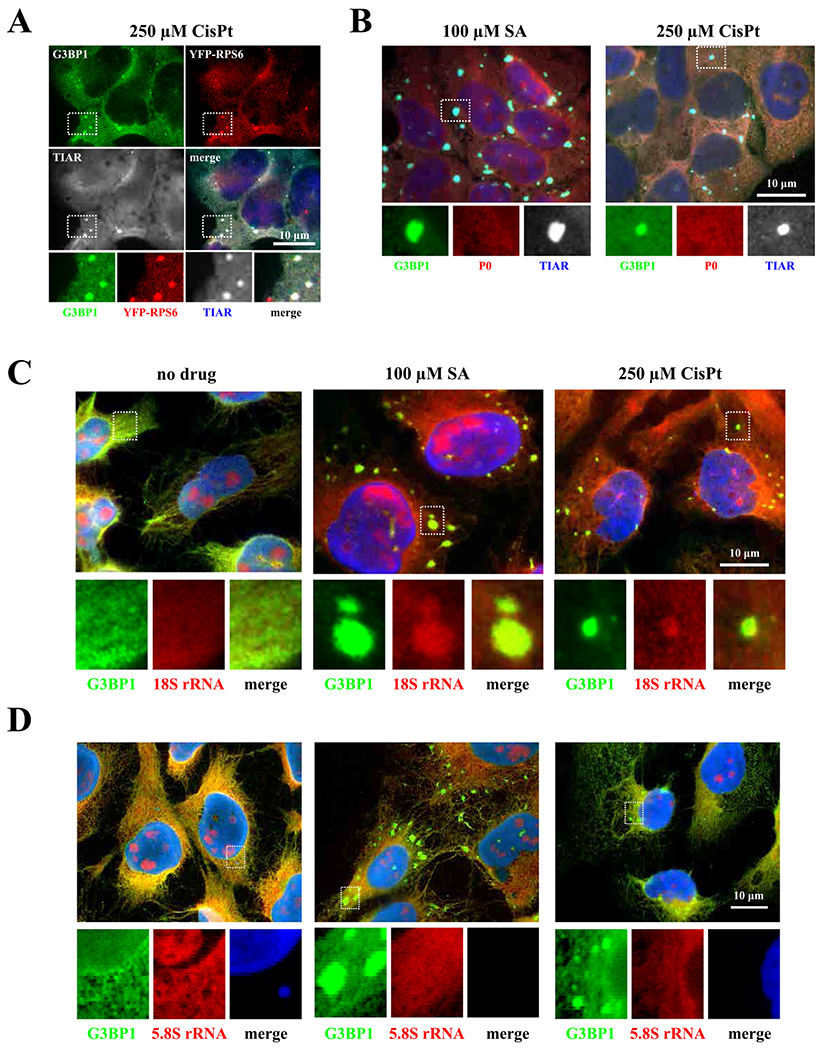
Detection of small (40S) and large (60S) ribosomal subunit components. (*A*) CisPt-induced foci contain ribosomal protein S6 (red) associated with 40S subunit. U2OS cells were stressed with cisplatin (CisPt, 250 μM) for 4 h. Cells were fixed and stained for G3BP1 (green), and TIAR (grey). YFP-RP6 was detected directly, without staining. (*B*) CisPt-induced foci do not contain P0 protein associated with 60 S subunit. U2OS cells were stressed with sodium arsenite (SA, 100 μM) for 1 h and cisplatin (CisPt, 250 μM) for 4 h (one population of U2OS cells were used as control – no drug). Cells were fixed and stained for three different proteins – G3BP1 (green), P0 (red), TIAR (blue/grey). Boxed region was shown enlarged with colors below each image. The size bar represents 10 μm. *(C)* CisPt-induced foci contain 18S rRNA. Cells were stressed with sodium arsenite (SA, 100 μM) for 1 h and cisplatin (CisPt, 250 μM) for 4 h (one population of U2OS cells were used as control – no drug). 18S rRNA was detected using FISH technique with ssDNA-oligo complementary to 18S rRNA. G3BP1 was consecutively stained and visualized. The size bar represents 10 μm. (D) CisPt-induced foci does not contain 5.8S rRNA. Cells were stressed with sodium arsenite (SA, 100 μM) for 1 h and cisplatin (CisPt, 250 μM) for 4 h (one population of U2OS cells were used as control – no drug). 5.8S rRNA was detected using FISH technique with ssDNA-oligo complementary to 5.8S rRNA. G3BP1 was consecutively stained and visualized. The size bar represents 10 μm.

It has been previously reported that diverse chemotherapy drugs can promote formation of SGs [26]. Using the SG-specific marker G3BP1, we tested whether platinum-based drugs such as CisPt, oxaliplatin (OxaPt) and carboplatin (CrbPt) also stimulate formation of G3BP1-positive cytoplasmatic foci as sodium arsenite (SA) and vinorelbine (VRB) in human osteosarcoma U2OS cells (Fig. 1A). Indeed, all tested platinum drugs induce formation of G3BP1-positive cytoplasmic foci in 20–40% of cells (Fig. 1A). To characterize these cytoplasmatic foci, we focused on CisPt as a representative member of platinum drugs. In contrast to SA- and VRB-induced SGs, CisPt-induced foci only contain some of the canonical SG markers, including TIAR and the small ribosomal subunit protein RPS6 (Fig. 3A) but completely lacking eIF3b, eIF4G (Fig. 1A). In the same time, CisPt foci were positive for the presence of 18S rRNA suggesting that they contain small ribosomal subunits (Fig. 3C). Just as canonical SA-induced SGs, large ribosomal subunit protein P0 are not found in CisPt induced foci (Fig. 3B). Using fluorescence in situ hybridization (FISH) to detect polyadenylated mRNAs [25], we failed to identify mRNAs (Fig. 2B) in CisPt-induced foci, in contrast to SA-induced SGs (Fig. 2B). In contrast with the failure to efficiently recruit polyadenylated mRNAs to CisPt foci (Fig. 2B), we observe a weak signal for poly (A)-binding protein (PABP) in CisPt-induced foci (Fig 2A). To determine whether CisPt foci resemble P-bodies (PBs), RNA granules closely related to SGs, we assessed Dcp1, a classical marker of PBs (Fig. 1C). We did not observe a colocalization of Dcp1 marker with G3BP1, proving that CisPt foci are not PBs and/or associated with PBs (Fig. 1C). In addition to U2OS osteosarcoma cells, CisPt potently induces G3BP1-positive, eIF4g and eIF3b- negative foci in other cancer cell lines (including HeLa (cervix), MES-SA (uterus) and SiHa (cervix)) under similar doses (Fig. S1).

Together, these data show that CisPt-induced foci contain some “canonical” SG components but lack others, most notably polyadenylated mRNAs and early translation initiation factors eIF3b and eIF4G (Figs. 1–3). Further analysis showed that CisPt foci are positive for some other known SG-associated proteins such as FXR1, TIA-1, TIAR, CAPRIN1 and UPF1 (Figs. S2 and S3).

As recruitment of specific signaling and apoptosis-related molecules into SGs is proposed to affect stress adaptation and survival of cells (reviewed in Ref. [19]), we next examined their localization after CisPt treatment (Fig. S2). p70 S6 kinase, TRAF2 and RSK2 localize to CisPt-induced foci similar to SA-induced SGs suggesting that although their protein composition is quite different, signaling molecules still shuttle into CisPt-induced foci similarly to SGs.

### 3.2. Cisplatin-induced foci are dynamically distinct from SGs

**Fig. 4.**
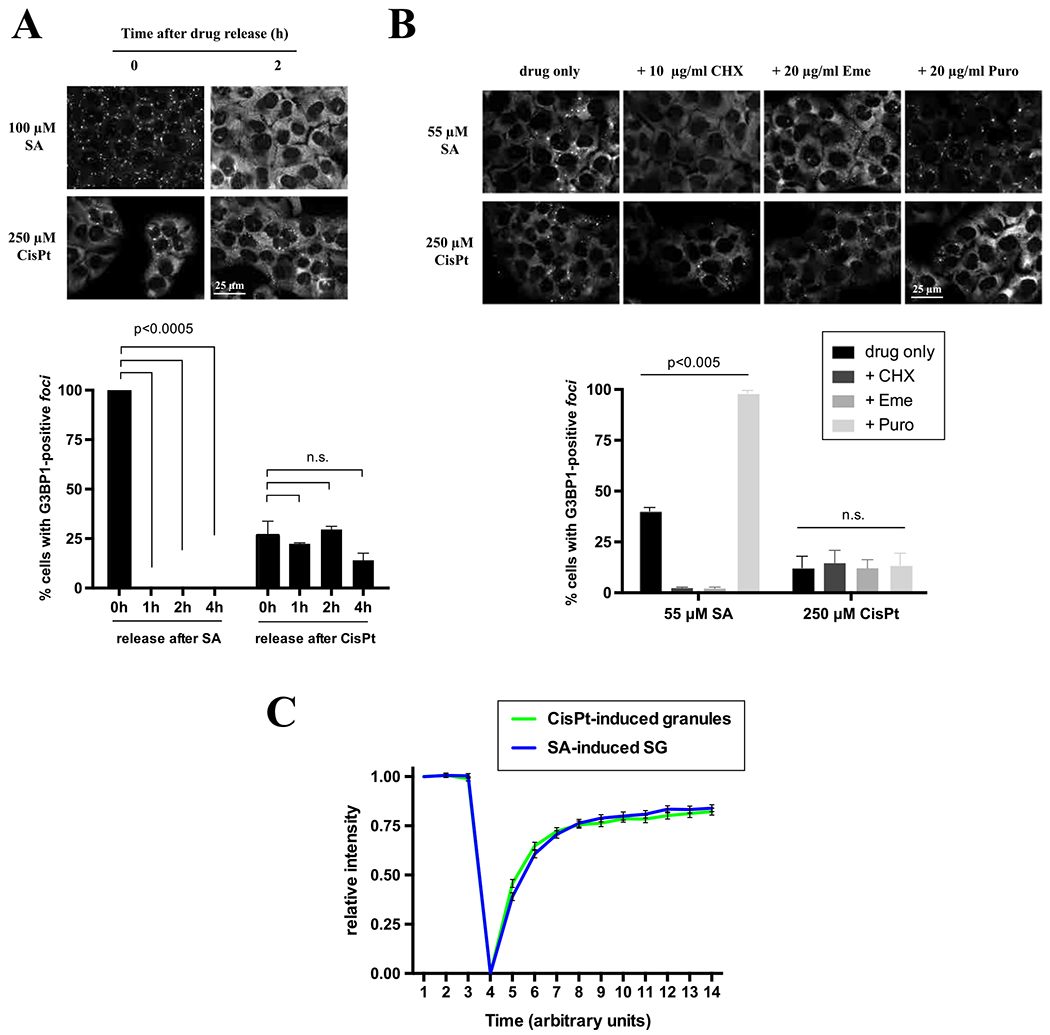
Features of CisPt-granules: dynamics and dependents on G3BP. (*A*) Dynamics of CisPt foci after stress relief. U2OS cells were treated either with sodium arsenite (SA, 100 μM) or cisplatin (CisPt, 250 μM) for 1 h and 4 h, respectively (negative control not shown). Then drug was removed form media and cells were incubated for additional 1, 2, and 4 h (control cells were fixed also directly after drug release – indicated as 0 h). Cells were fixed and stained for G3BP1 (green) and Hoechst (blue). Data were analyzed using the unpaired Student’s *t*-test, N = 3, and demonstrated on the graph. (*B*) Effects of translation inhibitors on CisPt foci formation. Cells were treated either with sodium arsenite (SA, 55 μM) or cisplatin (CisPt, 250 μM) for 1 h and 3.5 h followed by 1 h incubation with cycloheximide (CHX, 10 μg/ml), Emetine (Erne, 20 μg/ml) or Puromycin (Puro, 20 μg/ml). Cells were fixed and stained for G3BP1 (green) and Hoechst (blue). Data were analyzed using the unpaired Student’s *t*-test, N = 3, and demonstrated on the graph. (*C*) Quantification of CisPt-granules dynamics using FRAP technique. U2OS stable cell line GFP-G3BP1 was used. 3 frames were collected before bleaching and 20 after, all with an interval of 5 s in-between.

SGs are dynamic entities that assemble during stress and disassemble upon stress removal [29]. Reversible nature of SGs is considered to be the key attribute of SG-mediated stress adaptation where irreversible or less dynamic SGs (“pathological SGs”) are proposed to contribute to cell death. Therefore, we examined whether CisPt-induced foci dissolve like canonical SGs after removal of stress (e.g., when CisPt is washed out from treated cells). In contrast to the rapid disassembly of SA-induced SGs within 1 h, CisPt-induced foci are stable even four hours after stress removal (Fig. 4A) suggesting that they are more static and probably even irreversible (we could not monitor CisPt foci for longer periods of time after CisPt removal due to the CisPt-induced death of U2OS cells).

Further, SGs are in equilibrium with polysomes [29], actively translating fraction of ribosomes, and pharmacological manipulations that affect polysome dynamics also alter SG assembly and disassembly. Cycloheximide (CHX) and emetine (Eme) stall translating ribosomes causing polysome stabilization [25]. CHX and Eme treatment results in the rapid disassembly of SA-induced SGs (Fig. 4B) as reported before. However, these drugs failed to promote disassembly of CisPt-induced foci (Fig. 4B, CHX and Eme). Puromycin (Puro) is a translation inhibitor that collapses polysomes by premature termination and promotes SG assembly [25]. Puro treatment enhances the formation of SA-induced SGs but does not influence CisPt-induced foci (Fig. 4B, Puro). Together with data obtained from stress removal experiments (Fig. 4A), it suggests that CisPt foci are markedly different from canonical SGs.

SG components are also dynamic and in the move in and out of the granule [30]. The residing time of SG-associated proteins varies from seconds to minutes and some proteins reconstitute stable “core” while others constantly exchange between SG “shell” and surrounding cytosol. G3BP1 is one of canonical SG markers that is absolutely required for SG formation [31,32]. We hypothesized that observed changes in CisPt foci disassembly dynamics may be explained by changed shuttling abilities of G3BP1 (e.g., by some modifications of G3BP1 induced by CisPt treatment), Thus, we monitored the residence time of GFP-tagged G3BP1 using Fluorescence Recovery After Photobleaching (FRAP) in SA-induced SGs and CisPt-induced foci. In these experiments, the behavior of G3BP1 was similar in both SGs and CisPt-induced foci (> 90% recovery of the bleached signal occurred within 10 s) suggesting that G3BP1 rapidly shuttling in and out of CisPt-induced foci (Fig. 4C), and CisPt treatment does not affect G3BP1 ability to reversible associate with SGs.

**Fig. 5.**
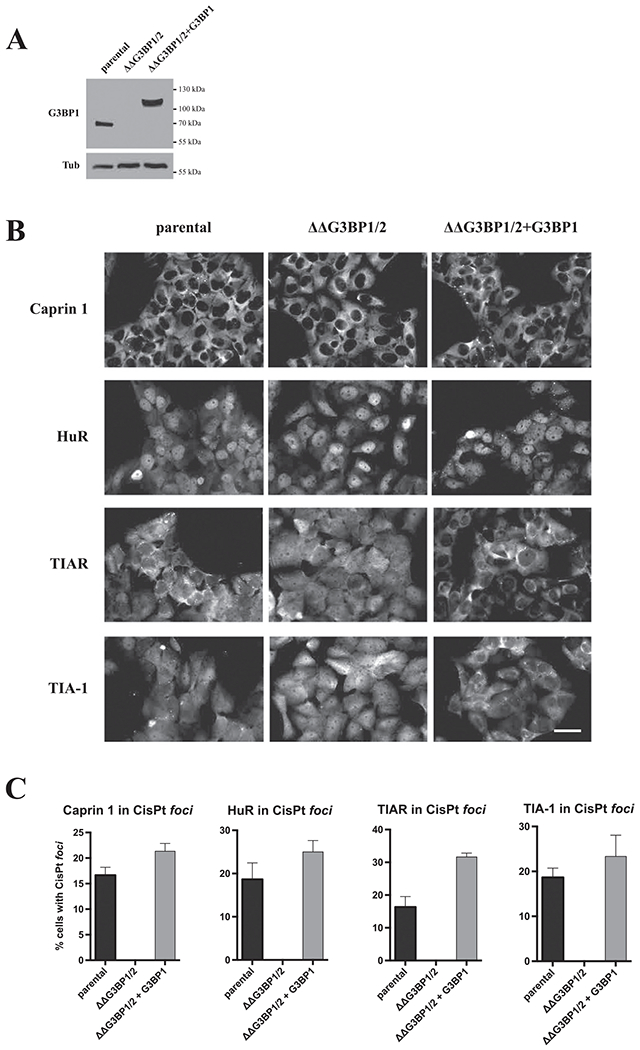
G3BP is absolutely required for recruitment of selected SG markers into CisPt foci. *(A)* Verification of ΔΔG3BP1/2 mutant and its rescue ΔΔG3BP1/2 + G3BP1 counterpart. All type of cells (parental U2OS, ΔΔG3BP1/2 mutant and ΔΔG3BP1/2 + G3BP1 rescue) were grown till 80% confluency. Then whole protein lysate was isolated and standard western blot against G3BP1 protein was executed. Tubulin β (Tub) was applied as a loading control. *(B)* Recruitment of CisPt foci in SG-competent (parental U2OS, ΔΔG3BP1/2 + G3BP1) and SG-incompetent (ΔΔG3BP1/2) U2OS cells. Classical marker of SG (Caprin1, HuR, TIAR and TIA-1) were applied. *(C)* Quantification of CisPt foci in SG-competent (parental U2OS, ΔΔG3BP1/2 + G3BP1) and SG-incompetent (ΔΔG3BP1/2) U2OS cells as shown in Fig. 5B.

G3BP is a protein critical for SG formation under most stresses [32]. We tested whether G3BP is also required for the assembly of CisPt-induced foci using U2OS cell line with genetic knockout of both G3BP proteins (ΔΔG3BP1/2) (Fig. 5A) by monitoring localization of SG-associated proteins that localize to CisPt foci [32]. Recruitment of SG markers Caprin 1, HuR, TIAR and TIA-1 into CisPt foci is completely abolished when compared to parental U2OS cells (Fig. 5B–C). Such recruitment defects are efficiently rescued by expression of G3BP1 (Fig. 5B–C, ΔΔG3BP1/2 + G3BP1). This suggests that G3BP is required for CisPt foci formation similarly to canonical SGs.

### 3.3. Cisplatin-induced foci are formed as a result of translation repression

Canonical SGs form when translation initiation is inhibited. To determine whether CisPt-induced foci are connected to translation initiation inhibition, we first examined whether CisPt treatment alters cellular translation using polysome profiling, a fractionation method of grossly assessing the overall translational state of cells.

**Fig. 6.**
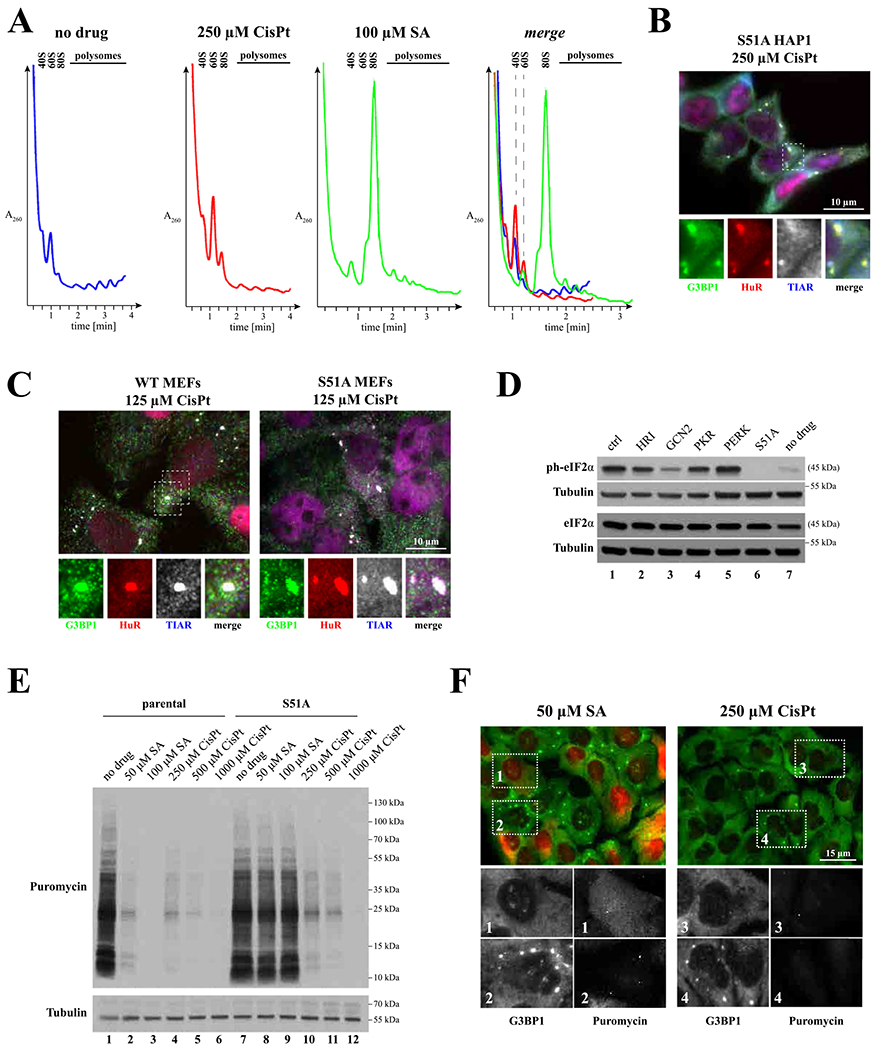
CisPt inhibits translation by promoting eIF2*α* phosphorylation. (*A*) Polysome profiles obtained from U2OS cells treated with cisplatin (CisPt, 250 μM) or sodium arsenite (SA, 100 μM) for 4 h and 1 h, respectively; as control, polysomes were isolated from untreated control cells (no drug). Polysome profiles lines were designated as follows: no drug – blue, 250 μM CisPt – red and 100 μM SA – green). *(B-C)* Formation of CisPt foci is independent of eIF2*α* phosphorylation. B: formation of CisPt foci in S51 HAP1 cells. C: formation of CisPt foci in WT and S51 mouse embryonic fibroblasts (MEFs). G3BP1, HuR and TIAR were used as markers. *(D)* Effect of CisPt on eIF2*α* phosphorylation. Parental HAP1 (ctrl), HAP1 variants with eIF2*α* kinase knockout genes (HRI, GCN2, PKR and PERK) or with eIF2*α* S51A mutation (S51A) were treated with CisPt (250 μM, 4 h). Untreated parental HAP1 cells (no drug) were used as controls. Lysates from treated and control cells were analyzed by western blotting using anti-phospho-eIF2*α* antibody (ph-eIF2*α*). Total eIF2*α* (eIF2*α*) and tubulin β (Tubulin) were used as loading controls. *(E)* Detection of translation activity in two HAP1 cells lines (parental, left part, and S51A, right part) treated with sodium arsenite (50 μM, 100 μM, SA), cisplatin (250 μM, 500 μM, 1000 μM, CisPt). No treated control (No drug) was used as control. U2OS cells were subjected to RiboPuromycylation to compare levels of basal translation. An anti-puromycin antibody (Puro) was used to visualize *de novo* synthesized proteins. Tubulin is a loading control. A representative image is shown (*n* = 3). *(F)* Detection of translation activity based on immunofluorescence technique. U2OS cells were treated with sodium arsentite (SA, 50 μM) or cisplatin (CisPt, 250 μM), fixed and stained with G3BP1 (green) to detect CisPt foci and anti-puromycin to monitor translation (red, shown as grey in boxed sub-image). The size bar represents 10 μm.

Polysome profiling indicates that CisPt promotes disassembly of polysomes and accumulation of monosomes and ribosomal subunits, although less potently than SA that was used as a control (Fig. 6A). While SA actively promotes formation of monosomes as previously reported, CisPt seems to cause accumulation of ribosomal subunits, although different explanations may exist.

Two main pathways regulate translation in response to stress, both targeting translation initiation: 1) control of initiator tRNA delivery to the ribosome by phosphorylation/dephosphorylation of eIF2α, and 2) mTOR- regulated binding of eIF4E-BPs to cap-binding protein eIF4E. In HAP1 cells [24], CisPt triggers robust eIF2α phosphorylation (ph-eIF2α, compare lanes 1 (ctrl) and 7 (no drug), Fig. 6D) but does not affect eIF2α protein levels (Fig. 6D, lower panel). CisPt-induced eIF2α phosphorylation is decreased by GCN2 kinase as GCN2 knockout cells (Fig. 6D, lane 3) but no other eIF2α kinases show decreased levels of ph-eIF2α (Fig. 6D, lanes 2–5). HAP1 cells bearing a non-phosphorylatable eIF2α mutant with Ser to Ala substitution at the position 51 (S51A) were used as control (Fig. 6D, lane 6). Further, puromycin labeling demonstrates that CisPt inhibits translation in both WT (Fig. 6E, compare lanes 4–6 with lane 1 (no treatment)) and eIF2α-S51A HAP1 cells (Fig. 6E, compare lanes 10–12 with lane 7 (no treatment)). This is in contrast to SA, which inhibits translation only in WT but not S51A HAP1 cells (Fig. 6E, lanes 1–3 and 7–9). This indicates that unlike SA, CisPt-induced translation repression can be stimulated by but not entirely dependent on eIF2α phosphorylation.

To determine whether phosphorylation of eIF2α is required for CisPt-induced foci assembly, we treated eIF2α-S51A HAP1 cells (Figs. 6B and S4) and eIF2α-S51A mutant mouse embryonic fibroblasts (MEFs, Fig. 6C) with CisPt. In both cases, CisPt-induced foci are formed suggesting that foci formation does not depend on eIF2α phosphorylation.

Also, it is previously reported that different chemotherapy drugs affect mTOR pathway to inhibit cellular translation and promote SG formation. As it is seen in Fig. S5, CisPt promotes some dephosphorylation of 4E-BP1 only at concentrations above 250 μM. In contrast, and in agreement with previous observations, SA does not cause 4E-BP dephosphorylation. Thus, 4E-BP1 dephosphorylation is not directly associated with CisPt foci formation in contrast to the previously reported effect of hydrogen peroxide (H_2_O_2_, [33]) and vinorelbine (VRB, [26]) on 4E-BP1, which were used as controls (Fig. S5). CisPt effects are also different from nitric oxide-induced inhibition of protein synthesis, which results from both phosphorylation of eIF2α and displacement of the eIF4F complex as a consequence of 4E-BP dephosphorylation [34]. Thus, CisPt triggers dephosphorylation of 4E-BP (at high concentrations) and phosphorylation of eIF2α to inhibit translation.

We further monitored effects of CisPt on cellular protein synthesis using alternative approach. Ribopuromycylation, a technique that directly assesses translation activity in cells, demonstrates that CisPt potently inhibits translation in both cells that assemble (Fig. 6F, box 4) and do not assemble (Fig. 4F, box 3) CisPt-induced foci. This is in contrast to SA, where translation inhibition and SG assembly are coupled (Fig. 4F, compare boxes 1 and 2).

### 3.4. Cisplatin suppresses formation of stress granules

**Fig. 7.**
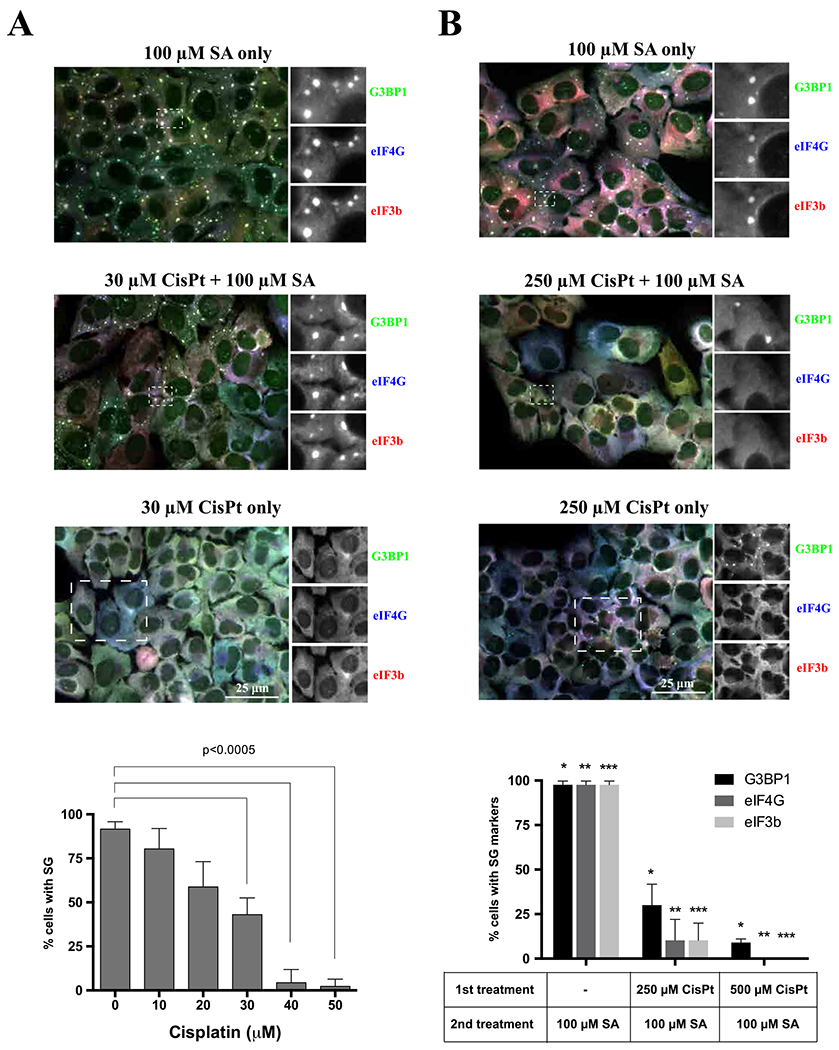
CisPt suppresses SG formation. (*A*) The formation of SA-induced stress granules was tested in U2OS cells in two populations of U2OS cells. Control population (untreated) and previously pretreated with increasing amount of cisplatin (0–50 μM) for 24 h. The cells from both populations were stressed with 100 μM sodium arsenite for 1 h. The upper image demonstrates population of U2OS cells stressed only with sodium arsenite (SA only, 100 μM), the middle image shows population of U2OS cells pretreated with cisplatin for 24 h and treated with sodium arsenite for 1 h (30 μM CisPt + 100 μM SA) and the lowest image shows cells treated only with CisPt; representative images. The cells were stained for canonical stress granules markers: G3BP1 (green), eIF4G (blue) and eIF3b (red). The main image was merged (RGB system). Boxed region was shown enlarged in grey corresponding to specific fluorescence channel as indicated. Data were analyzed using the unpaired Student’s *t*-test, N = 3, and demonstrated on the graph. (*B*) Control population (untreated) and previously pretreated with CisPt (no drug, 250 μM, 500 μM) for 3 h. The cells from both populations were stressed with 100 μM sodium arsenite for 1 h. The upper image demonstrates population of U2OS cells stressed only with sodium arsenite (SA only, 100 μM), the middle image shows population of U2OS cells pretreated with cisplatin for 3 h and treated with sodium arsenite for 1 h (250 μM CisPt + 100 μM SA) and the lowest images shows cells treated only with CisPt. The cells were stained for canonical stress granules markers: G3BP1 (green), eIF4G (blue) and eIF3b (red). The main image was merged (RGB system). Boxed region was shown enlarged in grey corresponding to specific fluorescence channel as indicated. Data were analyzed using the unpaired Student’s *t*-test, N = 3, and demonstrated on the graph. The size bar represents 10 μm.

Our data suggest that CisPt promotes formation of 40S-containing cytoplasmic foci by inhibition of cellular translation (Figs. 1 and 3). As 40S ribosomal subunits are core constitutes of SGs, we hypothesize that CisPt-induced accumulation of 40S subunits into these foci limits pool of ribosomes available for protein biosynthesis. Moreover, since polysomes are in equilibrium with SGs, we predicted that by decreasing the pool of actively translated ribosomes, CisPt will negatively affect formation of SGs. We pretreated cells with different concentrations of CisPt and then followed by a treatment with SA (Fig. 7). As can be judged by the recruitment of SG markers eIF4G and eIF3b, CisPt pre-treatment with low amounts of CisPt (10–50 μM, 24 h) causes dose-dependent, statistically significant, decrease of SG-positive cells (Fig. 7A). U2OS cells treated with higher concentrations of CisPt (250 μM, Fig. 7B) readily demonstrate significantly reduced SG formation at shorter times (1–3 h). Thus, treatment with CisPt promotes formation of CisPt foci that reduce abilities of cell to promote SG formation in response to stress.

## 4. Discussion

Cisplatin plays a key role in cancer chemotherapy where it is highly effective against a variety of solid tumors [1,3]. Historically, DNA is generally considered as a major biological target of CisPt. Upon entering the cell, CisPt is activated through a serious of spontaneous aquation reactions resulting in the generation of a powerful electrophile [35,36]. The monoaquated form represents as a highly reactive species, which formation is regulated by the interaction with a number of intracellular nucleophiles. These endogenous nucleophiles such as proteins, glutathione or methionine contribute to the intracellular inactivation of CisPt thus modulating its bioactivity.

This simple model where DNA damage underlines CisPt cytotoxicity is challenged by other studies. They suggest that CisPt cytotoxicity originates from multiple sources besides DNA damage-mediated [37], e.g. by targeting RNA metabolism by interference with telomerase functions [17], or inhibition of protein synthesis, transcription and splicing [38]. Moreover, experiments on enucleated cells demonstrated that CisPt-induced cytotoxicity does not involve DNA damage [11]. In agreement with it, only limited amount of intracellular CisPt is covalently bound to DNA, and there is no linear correlation between the extent of DNA platination and its toxicity to cells [12]. Thus, the ability of CisPt to induce nuclear DNA damage per se is not sufficient to explain its high degree of effectiveness on highly proliferative cancer cells nor the cytotoxic effects exerted on normal, post-mitotic tissues.

Our analysis reveals effects of CisPt on different aspects of RNA metabolism such as protein synthesis and RNA granule formation. Although we do not directly link these effects to CisPt-induced cytotoxicity, such effects may exist. Our data suggest that CisPt strongly affects RNA metabolism by modulation of common stress responses acting on post-transcriptional level. We show that CisPt potently inhibits cellular translation (Fig. 6E–F). This CisPt-mediated inhibition of protein synthesis may be mediated by partial inactivation of mTOR leading to dephosphorylation of 4E-BP1 (Fig. S5) and/or by phosphorylation of eIF2α via activation of the GCN2 kinase (Fig. 6D), although the relative contribution of these signaling pathways to CisPt effects on translation needs to be further investigated. Both mTOR inactivation and phosphorylation of eIF2α lead to the inhibition of translation initiation and partial reduction of polysomes (Fig. 6A). We think that activation of both pathways is likely to be a consequence of mTOR and GCN2 sensing reactive oxygen species induced by CisPt-mediated damage of mitochondrial species [39] rather than by direct interaction with the drug.

Inhibition of translation initiation is commonly coupled with formation of SGs [21]. SGs form in response to various extra- and intra-cellular insults and aim on stress adaptation [40]. SGs can promote viability by several mechanisms, which serve to conserve and redirect cellular energy towards pro-survival strategies. Several chemotherapy agents have been previously reported to promote SG formation. In contrast to these drugs, CisPt induces formation of unique cytoplasmic foci that are distinct from SGs, although share with them some canonical components such as 40S ribosomal subunits, markers G3BP1, TIAR or PABP (Figs. 1 and 2) as well as some signaling molecules (Fig. S2). CisPt foci are also different from P bodies (Fig. 1C), other well-known cytoplasmic RNA granules. Albeit the presence of 40S subunits, CisPt foci lack poly(A) mRNAs (Fig. 2B) that can explain the absence of initiation factors eIF3b and eIF4G (Fig. 1A). It is important to note that although CisPt promotes phosphorylation of eIF2α, it promotes CisPt formation in phospho-eIF2α-independent manner (Fig. 6B–C). The protein composition of SGs may also be important in predicting the aggressiveness of cancer in patients [41].

Another striking difference of CisPt foci to SGs is that their formation is largely irreversible (Fig. 4A–B). While SGs are quickly dissolving after stress relief, CisPt foci are static and long lived after drug removal (Fig. 4A). In agreement with static nature of CisPt foci, pharmaceuticals manipulations with polysomes, the fraction of ribosomes that are in dynamic equilibrium with SGs, do not affect formation of these foci (Fig. 4B). In the same time, formation of CisPt foci and SGs is absolutely dependent on the activities of G3BP1, which dynamically associates with 40S subunits and promotes SG condensation. G3BP1 shuttles on and off CisPt foci with kinetics similar to observed with SGs (Fig. 4C), and regulates recruitment of other SG markers into CisPt foci (Fig. 5C). All these data suggest that CisPt foci are both distinct from and related to SGs in terms of their composition and molecular mechanisms of their assembly.

Another possible mechanism of CisPt foci formation is its ability to bind ribosomes directly. The study by the Polikanov laboratory demonstrates that CisPt directly binds to ribosomes and modifies their functional centers such as the mRNA-channel and the GTPase center [42]. By binding to these centers, CisPt interferes with mRNA-ribosome interactions resulting in impaired mRNA translocation and inhibition of protein synthesis. If mechanisms of Cis-Pt binding to ribosomes are conserved between archaea and higher eukaryotes, we propose that CisPt also bind mammalian ribosomes and/or their subunits. By binding to the ribosomes, CisPt inactivates them in a manner that promotes accumulation of 40S subunits into CisPt foci. As 40S subunits are core components of SGs, we predicted that pre-treatment of cells with CisPt would limit available pool of 40S subunits and suppress SGs formation. In agreement with such prediction, incubation of cells with CisPt directly impact their ability to assemble SGs in both time- and concentration-dependent manners (Fig. 7A–B).

The finding that CisPt inhibits SG formation may also contribute to observed CisPt cytotoxicity. As SGs are pro-survival, suppression of their formation contributes to cell death, especially under stress conditions. Although this hypothesis still need examination, we propose here that in rapidly proliferating cancer cells, suppression of SGs may contribute to CisPt-mediated cell death together with other mechanisms such as DNA damage. However, as CisPt also accumulates in specific cells (nephrons, inner ear cells), which are not cancerous, inhibition of SG formation and protein synthesis may be dominant mechanisms underlying CisPt cytotoxicity.

## Supplementary Material

Supplementary Material
