# Supplementary figures and images for: Translation inhibition and suppression of stress granules formation by cisplatin
Source: Biomed Pharmacother. Author manuscript; Available in PMC 2022 Jan 21. (PMC8782064; doi:10.1016/j.biopha.2021.112382)

# SiHa

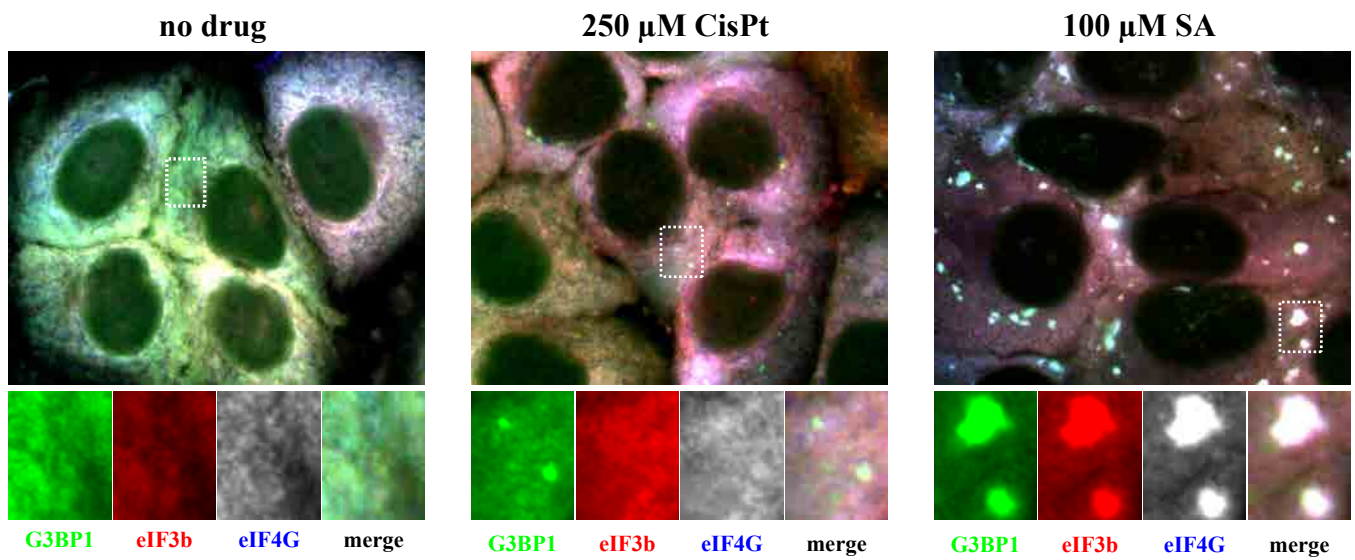

# MES-SA

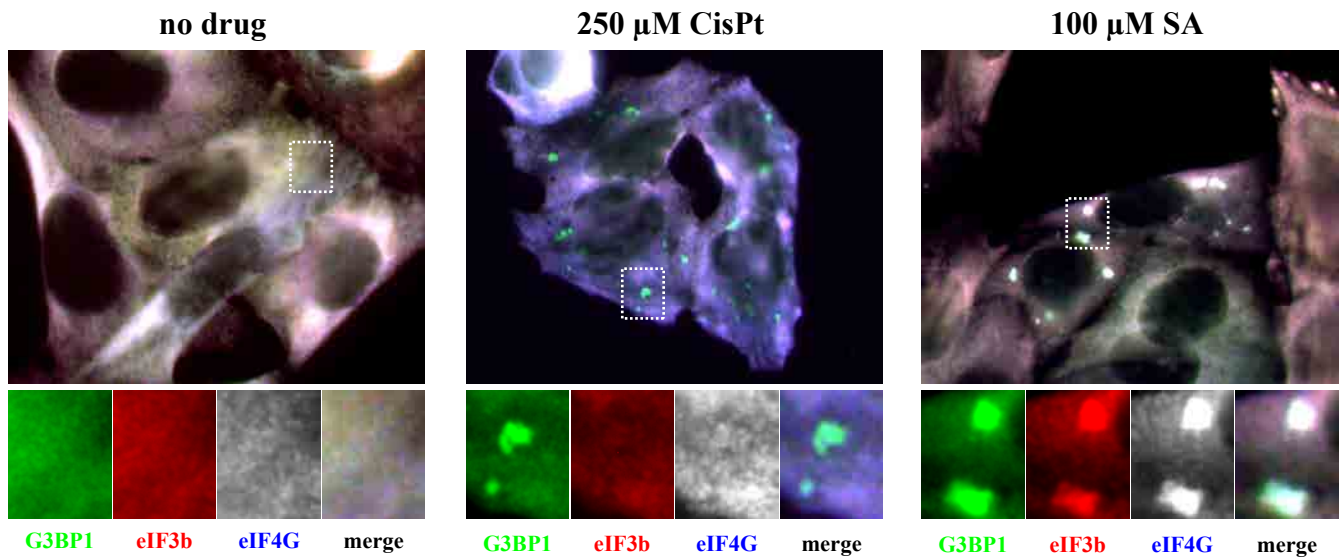

# HeLa

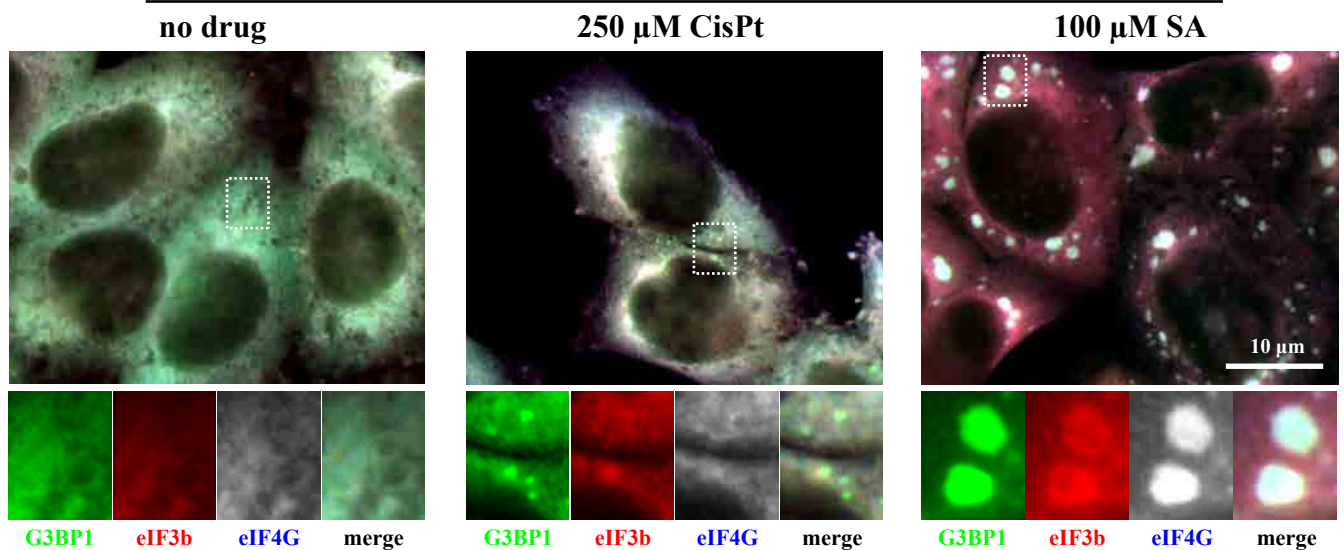

**Figure S1**

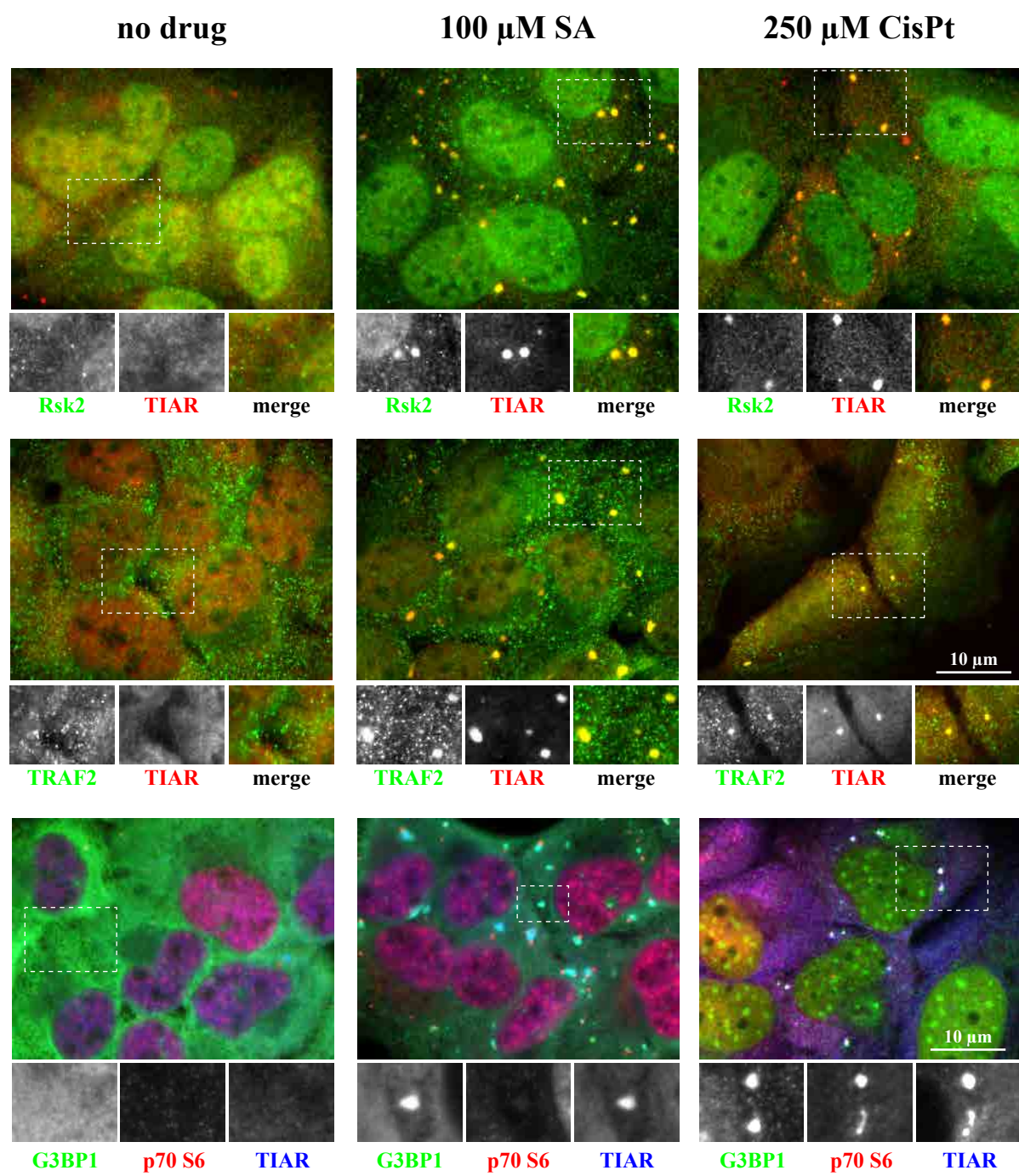

**Figure S2**

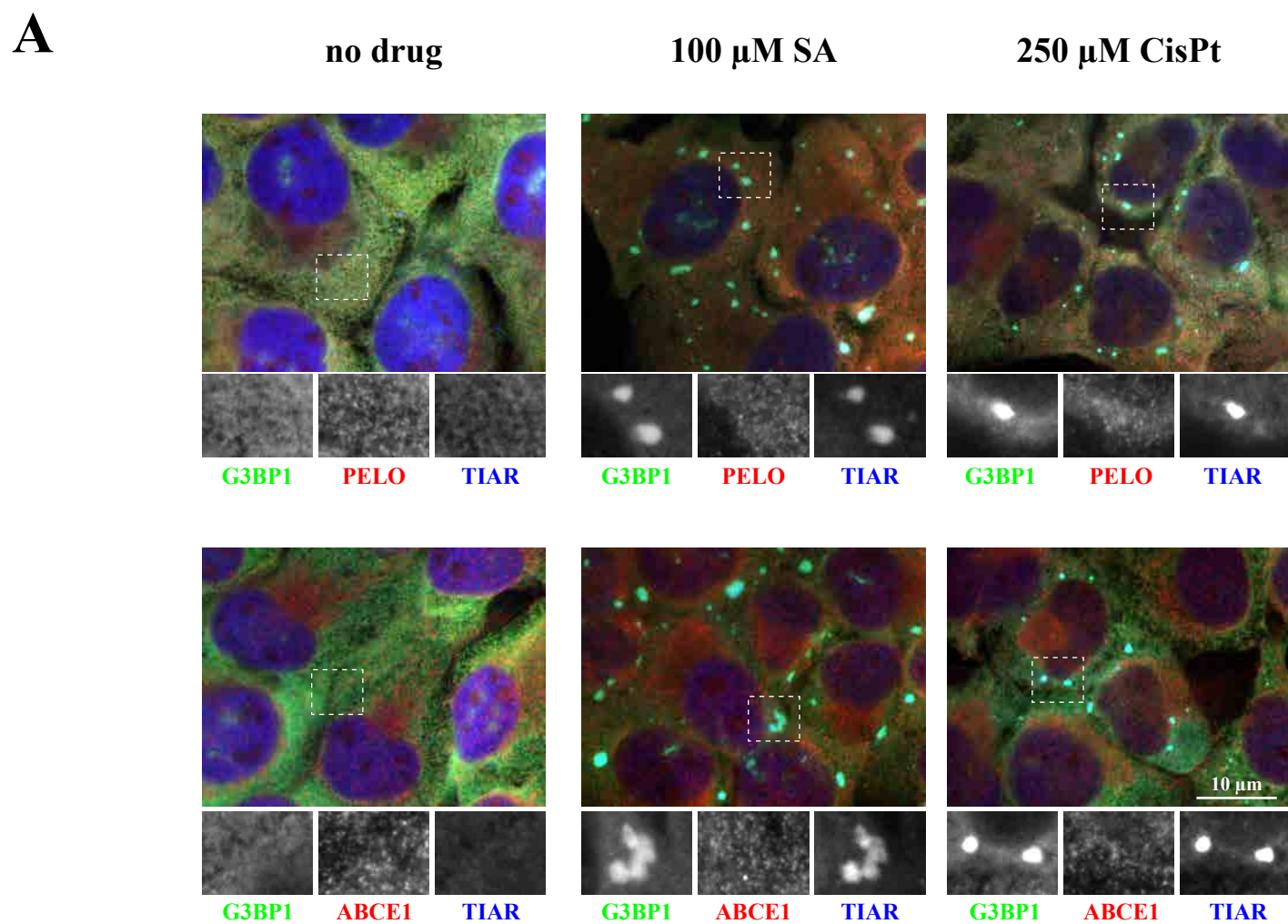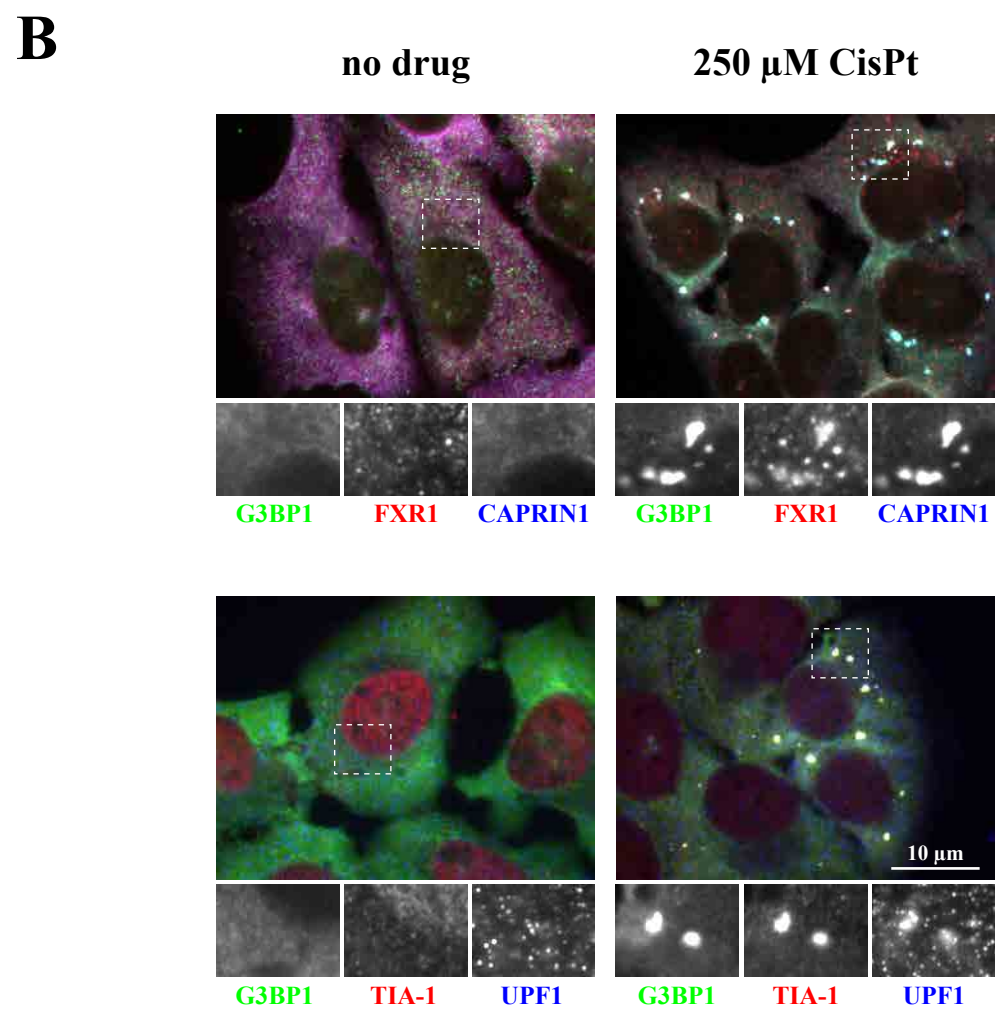

**Figure S3**

**A**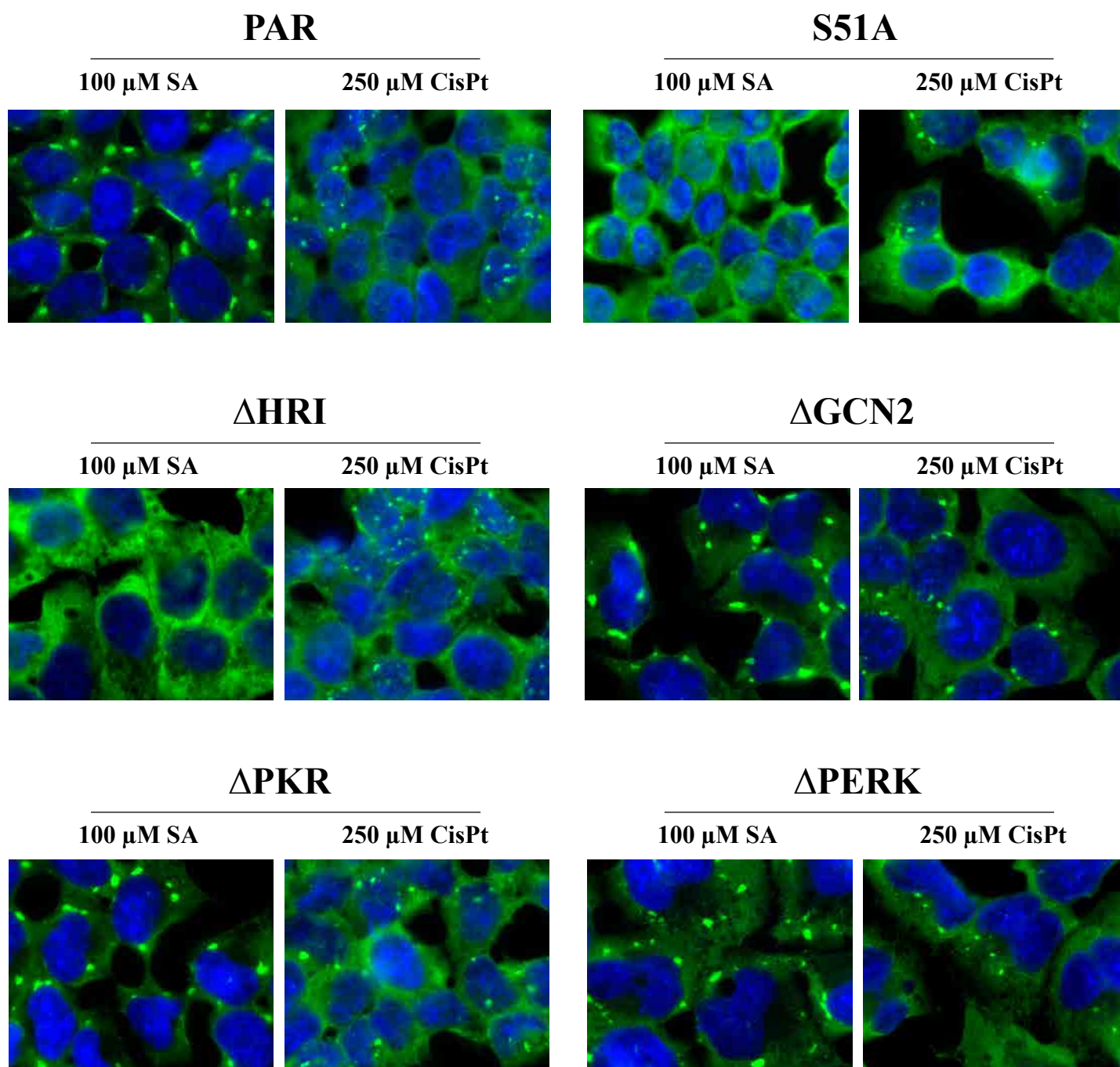**B**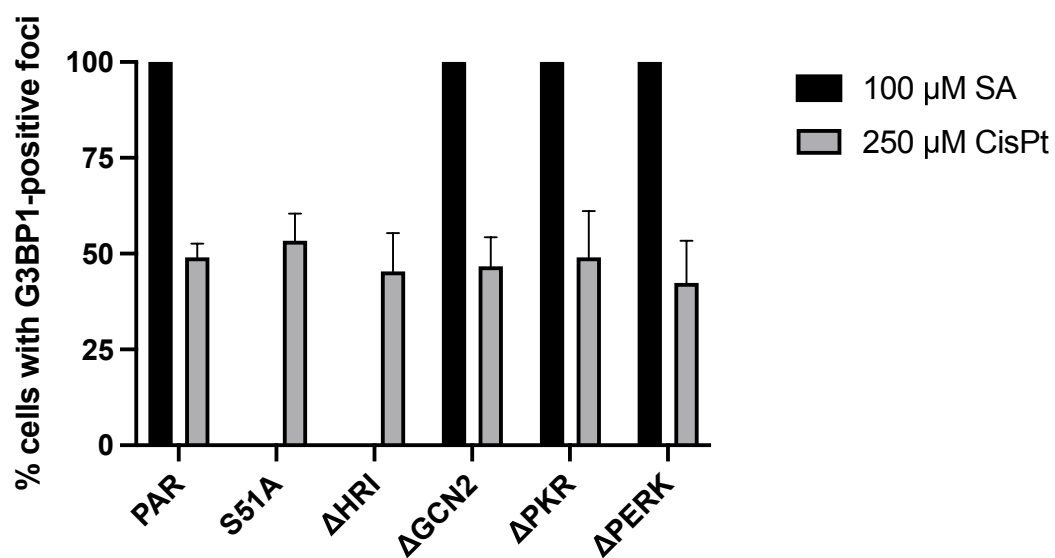**Figure S4**

**A**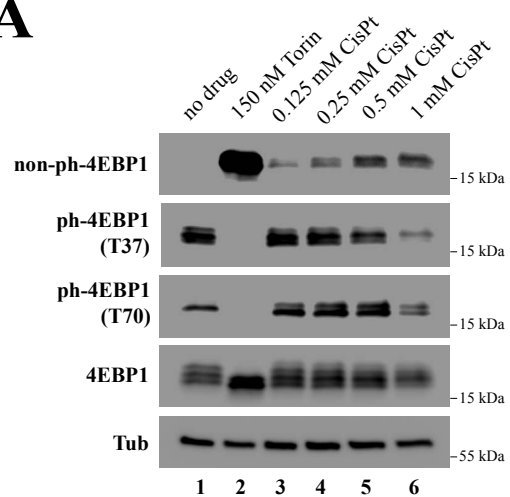**B**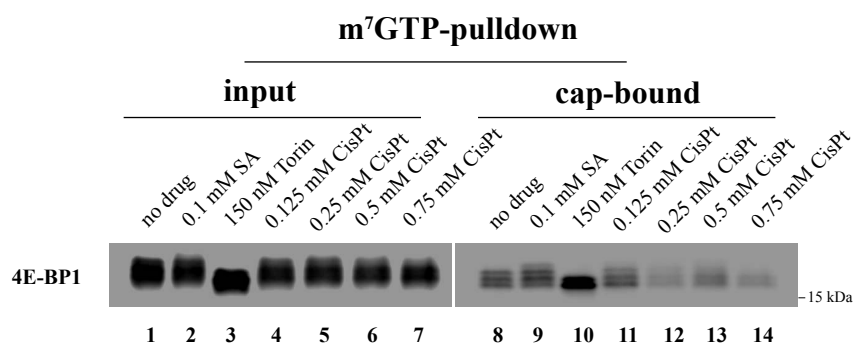**Figure S5**

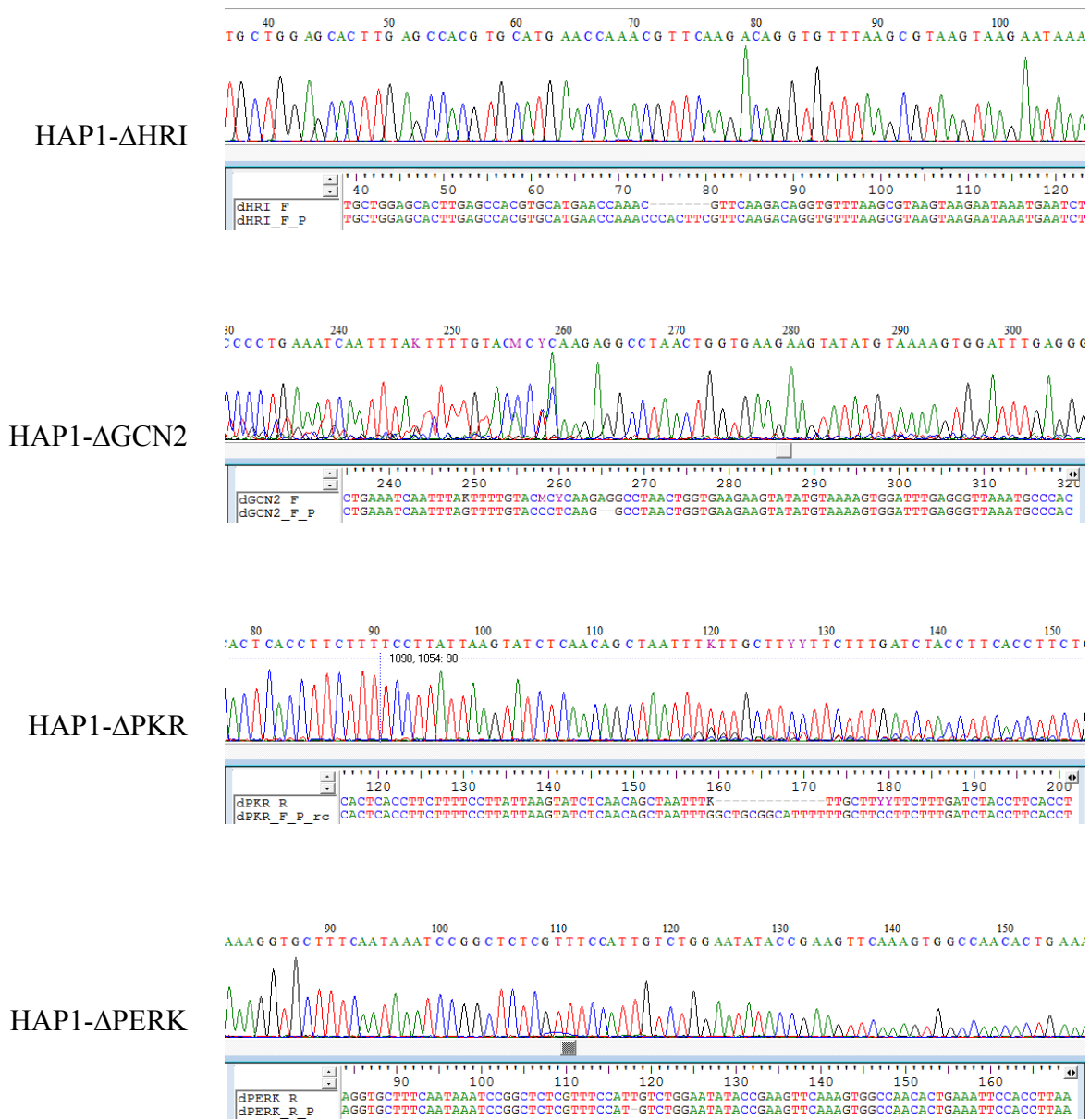

**Figure S6**

Supplement: Supplementary Material [file NIHMS1769611-supplement-Supplementary_Material.pdf]
